# The Correlation of Surfactant Concentrations on the Properties of Mesoporous Bioactive Glass

**DOI:** 10.3390/ma9010058

**Published:** 2016-01-19

**Authors:** Shao-Ju Shih, Yu-Chien Lin, Leon Valentino Posma Panjaitan, Dyka Rahayu Meyla Sari

**Affiliations:** Department of Materials Science and Engineering, National Taiwan University of Science and Technology, 43, Section 4 Keelung Road, Taipei 10607, Taiwan; M10304306@mail.ntust.edu.tw (Y.C.L.); M10004806@mail.ntust.edu.tw (L.V.P.P.); 10204804@mail.ntust.edu.tw (D.R.M.S.)

**Keywords:** mesoporous bioactive glass, morphology, surface area, spray pyrolysis, formation mechanism

## Abstract

Bioactive glass (BG), a potential biomaterial, has received increasing attention since the discovery of its superior bioactivity. One of the main research objectives is to improve the bioactive property of BGs; therefore, surfactant-derived mesoporous bioactive glasses (MBGs) were developed to provide a high specific surface area for achieving higher bioactivity. In this study, various concentrations of typical triblock F127 surfactant were used to manipulate the morphology, specific surface area, and bioactivity of MBG particles. Two typical morphologies of smooth (Type I) and wrinkled (Type II) spheres were observed, and the population of Type II particles increased with an increase in the surfactant concentration. A direct correlation between specific surface area and bioactivity was observed by comparing the data obtained using the nitrogen adsorption-desorption method and *in vitro* bioactive tests. Furthermore, the optimal surfactant concentration corresponding to the highest bioactivity revealed that the surfactant aggregated to form Type II particles when the surface concentration was higher than the critical micelle concentration, and the high population of Type II particles may reduce the specific surface area because of the loss of bioactivity. Moreover, the formation mechanism of SP-derived MBG particles is discussed.

## 1. Introduction

SiO_2_, CaO, and P_2_O_5_-based bioactive glasses (BGs) have been used as bioactive materials in drug carriers [1,2], dental sealing, and [3] bone implants [4,5] because of their superior bioactivity, which was first proposed by Hench *et al.* in 1971 [6]. The bioactivity of a material is its ability to form hydroxyl apatite (HA) after it is embedded in the human body [7,8] or immersed in a simulated body fluid (SBF) [9]. HA, the main inorganic bone component, provides the specific biological response of bonding at the tissue-BG interface [10]. Because bioactivity is crucial, improving the bioactive behavior of BGs has attracted considerable attention.

For improving bioactive behaviors, a suitable chemical composition and high specific surface area of BGs are essential, and two methods are available for achieving this goal. First, the basis of the bone-bonding property is the chemical reaction of BGs in body fluids. This reaction involves five steps: (i) the formation of silanol (SiOH); (ii) the loss of soluble silica and formation of SiOH; (iii) polycondensation of SiOH; (iv) the formation of an amorphous calcium phosphate layer; and (v) the crystallization of the HA layer [11]. More details can be found in the study by Hench *et al.* [11,12]. They claimed that the kinetics of these reaction stages depend on the BG composition [11]. For example, the first and second stages are associated with the SiOH formation, which is essential for the growth of HA [11]. A previous study reported that non-bridging oxygen (NBO) groups exchange H^+^ or H_3_O from the surrounding SBFs to increase the SiOH formation [11]. Consequently, in BGs, Ca acts as a modifier to reduce the degree of connectivity in the glassy structure for increasing the population of NBO groups. Therefore, a higher Ca concentration (e.g., 45S5 glass [12]) provides more NBO groups, leading to a high dissolution rate and high bioactivity.

The other approach for achieving a high bioactivity is to increase the HA formation rate by maximizing the specific surface area. For example, Mačković *et al.* reported that the 45S5 BG nanoparticles exhibit a higher surface area and a faster HA formation (immersion in an SBF for 1 day) than micron-sized particles do (immersion in an SBF for 3 days) [13]. Furthermore, Yan *et al.* used surfactants to prepare a well-ordered mesoporous bioactive glass (MBG) with a high specific surface area [14], and, consequently, numerous studies have used the mesoporous structure to obtain a high bioactivity (HA formation after immersion in an SBF 4–8 h) [15,16]. The mesoporous structure has become a requirement for obtaining BG materials.

Because the mesoporous structure is crucial, two methods, sol-gel [14,15,16] and spray pyrolysis (SP) [17], have been used to fabricate MBGs. Compared to sol-gel, SP is faster and more continuous [18]. Although SP-derived MBGs have the potential for mass production, inhomogeneous particles (smooth and wrinkled spheres [17]) have been observed in SP-derived MBGs. This inhomogeneity may result in the loss of specific surface area, and, based on our research, studies that describe the relationship between morphology and specific surface area for SP-derived MBG particles are rare. For a mesoporous structure, surfactants play a crucial role to form micelles as a template for conducting pore formation [19]. When the surfactant concentration in an aqueous solution is low, surfactants are located as separate molecules in the water and are unable to form micelle channels. When the surfactant concentration increases and reaches a critical value, the surfactant molecules come together to form micelles, and this concentration is called the critical micelle concentration (CMC) [19]. The uses of surfactant for the MBGs, especially when the concentration is higher than the CMC, were reported by Yan *et al.* [20] and Soulie *et al.* [21]. However, increasing the concentration further causes the surfactant molecules to aggregate and produce elongated tubes and stacked lamellae at the second and third CMCs, respectively [22,23]. In addition, Myers *et al.* predicted that the morphologies of the surfactant at high concentrations are spherical, rod-shaped, and have a flexible bilayer structure, planar extended bilayers, and reversed micelles [24]. These morphologies may deteriorate the compacted mesoporous structure and reduce the specific surface area. Therefore, the surfactant concentration is related to the formation of mesoporous structures and affects the specific surface area and bioactivity of MBG particles.

In this study, the common triblock surfactant, F127 (Pluronic F-127, EO_106_PO_70_EO_106_, where EO is polyethylene oxide and PO is polypropylene oxide (Sigma-Aldrich, Ludwigshafen, Germany) was used to produce the mesoporous structure of BG powder, and various surfactant concentration-treated MBG powders were synthesized and characterized to examine the relationship among surfactant concentration, specific surface area, and bioactivity. First, X-ray diffraction (XRD) was used to characterize the phase compositions of these MBG powders. Second, scanning electron microscopy (SEM) and transmission electron microscopy (TEM) were used to observe their surface structures and geometries. In addition, particle size distributions and pore sizes were acquired using a couple of SEM and TEM images, respectively. Third, the specific surface areas of the powders were determined using the nitrogen adsorption-desorption method (Brunauer-Emmett-Teller (BET) method) and were compared with particle morphologies. Finally, for *in vitro* bioactive tests, the Fourier transform infrared reflection (FTIR) spectrophotometer (Varian Inc., Palo Alto, CA, USA) was used to characterize the powders after they were soaked in an SBF for 1 day, and the intensity of P-O bonding of the various MBG powders were acquired to determine their bioactivity [7].

## 2. Results

### 2.1. Phase Composition

**Figure 1:**
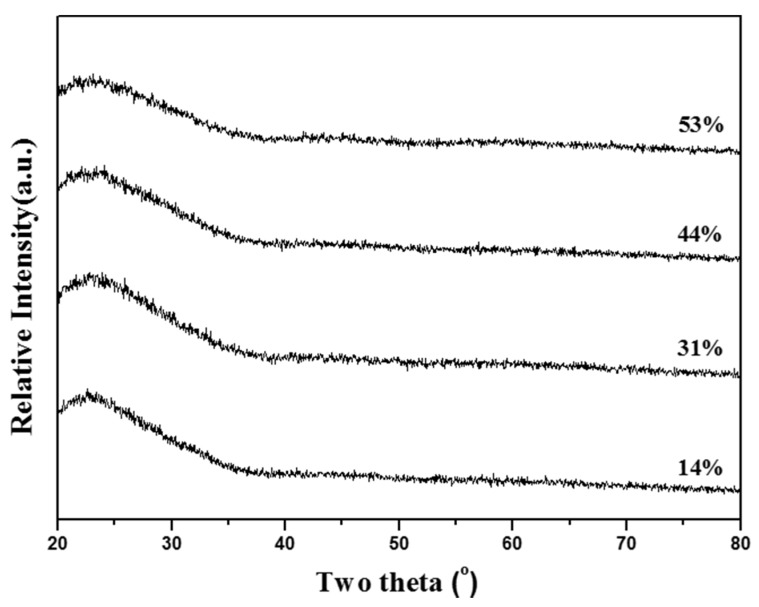
XRD patterns of MBG particles prepared using various surfactant concentrations.

Figure 1 shows the phase composition of four MBG powders prepared using distinct F127 (surfactant) concentrations of 14, 31, 44, and 53 wt %. Only a broad band between 2θ angles of 20.0° and 37.0° was detected in each XRD spectrum, indicating that all MBG powders were amorphous and that surfactant concentration did not affect the phase composition of MBG powders.

### 2.2. Morphology

**Figure 2:**
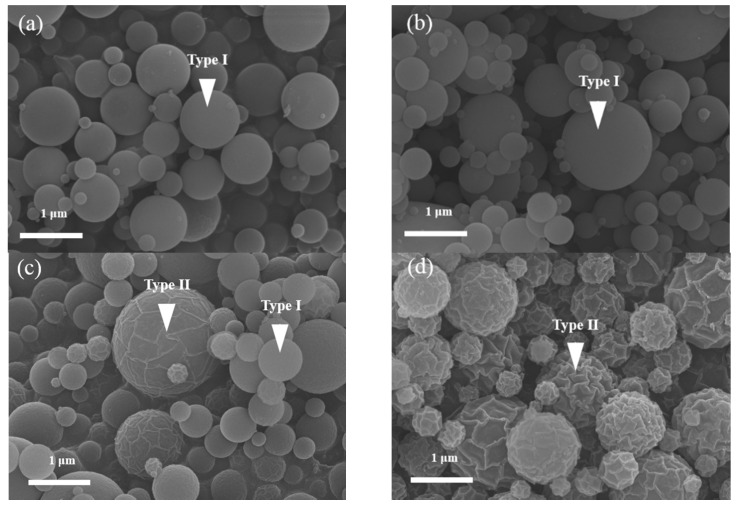
SEM images of MBG particles prepared using various surfactant concentrations of (**a**) 14 wt %; (**b**) 31 wt %; (**c**) 44 wt %; and (**d**) 53 wt %.

**Figure 3:**
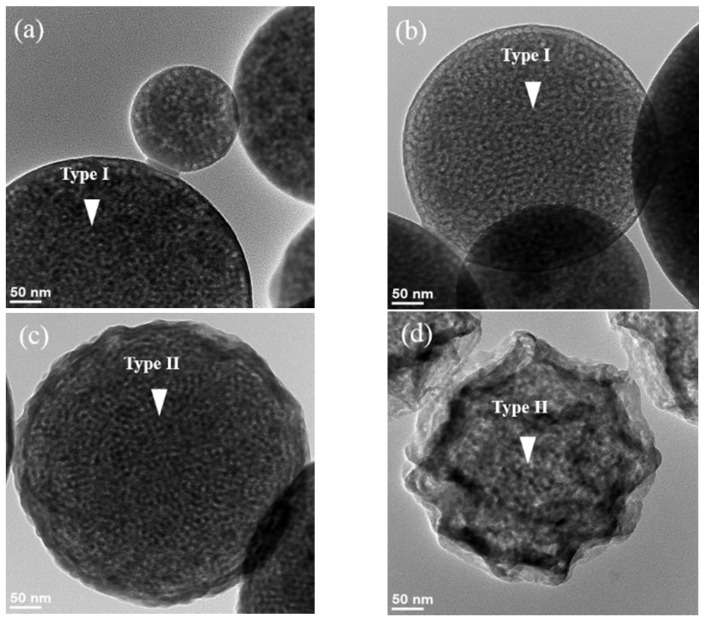
TEM images of MBG particles prepared using various surfactant concentrations of (**a**) 14 wt %; (**b**) 31 wt %; (**c**) 44 wt %; and (**d**) 53 wt %.

For a detailed morphology, the surface structure and geometry of the MBG powders were characterized using SEM and TEM, respectively. Figure 2 shows the SEM micrographs of four MBG powders prepared using different surfactant concentrations. A smooth spherical surface structure was observed for 14 wt % F127-treated MBG particles (Figure 2a). In addition, the same smooth spherical structure was observed for 31 wt % F127-treated MBG particles (Figure 2b). However, for 44 and 53 wt % F127-treated MBG particles, the SEM micrographs reveal two surface structures, smooth and wrinkled spheres, of MBG particles (Figure 2c,d). Furthermore, the particle geometries of MBG powders were investigated using TEM (Figure 3). Figure 3a shows bright and dark regions within a particle, and these bright and dark regions corresponded to pores and walls [25], thus proving the mesopore formation within the 14 wt % F127-treated MBG particles. In addition, the same geometry was observed for the 31 wt % F127-treated MBG particles (Figure 3b). However, for 44 and 53 wt % F127-treated MBG particles, both a mesoporous structure and an irregular surface were observed (Figure 3c,d). Furthermore, from the TEM images, the average values and standard deviations of pore size for 14, 31, 44, and 53 wt % F127 were 6.9 ± 1.3, 7.4 ± 1.5, 7.1 ± 1.4, and 5.5 ± 1.3 nm, respectively. By combining the SEM and TEM micrographs, two morphologies, smooth mesoporous (Type I) and wrinkled mesoporous (Type II), were observed. This result suggests that MBG particles treated with lower F127 concentrations (14 wt % and 31 wt % F127-treated MBG particles) exhibited only Type I morphology, whereas those treated with higher F127 concentrations (44 and 53 wt % F127-treated MBG particles) exhibited both Type I and Type II morphologies. This implies that the MBG morphology is a function of F127 concentration. In summary, particle morphologies were constructed using surface structure and geometry, which were observed using SEM and TEM.

### 2.3. Particle Size Distribution and Specific Surface Area

**Figure 4:**
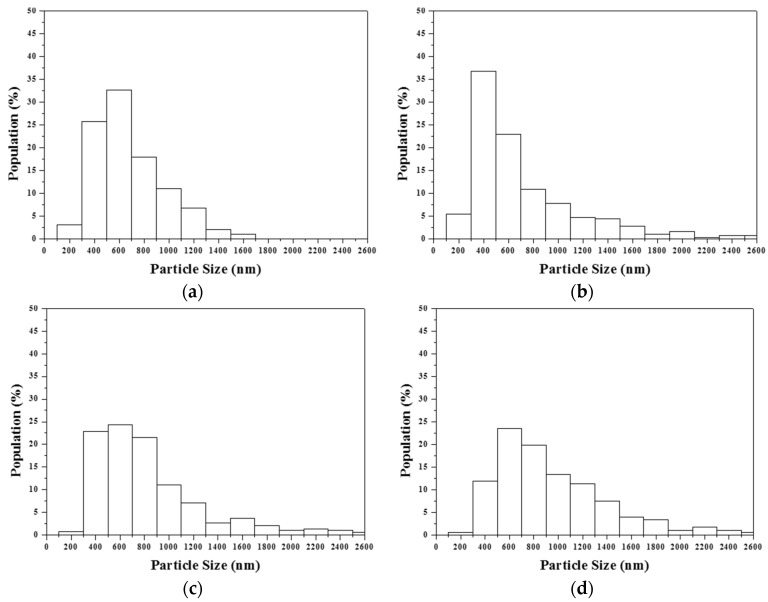
Particle size distributions of MBG particles prepared using various surfactant concentrations of (**a**) 14 wt %; (**b**) 31 wt %; (**c**) 44 wt %; and (**d**) 53 wt %.

The particle size distributions of various F127-treated MBG powders are shown in Figure 4. The average particle sizes and standard deviations of the 14 wt %, 31 wt %, 44 wt %, and 53 wt % F127-treated MBG powders were 579 ± 275, 878 ± 675, 942 ± 474, and 1119 ± 624 nm, respectively. This result revealed that the particle size increased with an increase in the surfactant concentration. The BET measurements showed that the specific surface areas of the 14 wt %, 31 wt %, 44 wt %, and 53 wt % F127-treated MBG powders were 104.2 ± 19.8, 258.3 ± 21.1, 338.9 ± 12.3, and 296.6 ± 12.6 m^2^/g, respectively. The BET data showed that the specific surface area had the maximum value of 338.9 m^2^/g when the F127 concentration was 44 wt %. However, when the surfactant concentration was increased to 53 wt %, the specific surface area decreased to 296.6 m^2^/g.

### 2.4. In Vitro Bioactive Test

**Figure 5:**
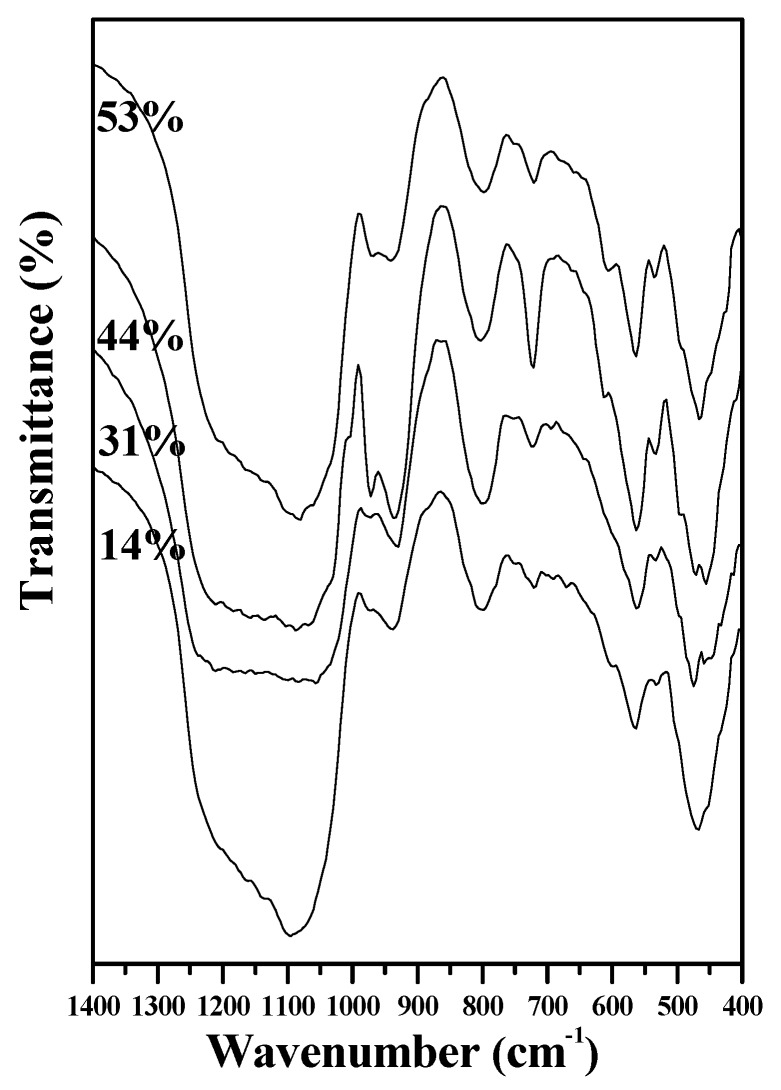
FTIR patterns of various surfactant concentration treated MBG particles immersed in SBF for 1 day.

The characterization of the HA crystallinity of SBF-immersed BGs is commonly used to determine the corresponding bioactivities [11]. Figure 5 shows the FTIR patterns of 14 wt %, 31 wt %, 44 wt %, and 53 wt % F127-treated MBG powders before and after immersion in an SBF for 1 day. The peaks at 1095 and 482 cm^−1^ are assigned as a Si-O-Si stretching vibration and a Si–O–Si bending mode, respectively [26,27]. The peaks at 598 and 566 cm^−1^ are assigned as P–O bending vibrations in PO_4_ tetrahedra [28]. The bioactivity is determined by obtaining a ratio of peak intensities from the FTIR patterns. I_1_ refers to the intensity of P–O bending vibration around 566 cm^−1^, whereas I_2_ refers to the intensity of Si–O–Si bending vibration at 482 cm^−1^. The I_1_/I_2_ value corresponds the amount of HA: the higher the I_1_/I_2_ the larger amount of HA. Note that all MBG powders exhibited an amorphous structure before immersion in an SBF (Figure 1). After immersion in an SBF for 1 day, bioactivity was directly related to the HA formation rates after immersion in an SBF and in turn to the HA amount after the same SBF immersion time. The I_1_/I_2_ values of 14, 31, 44, and 53 wt % F127-treated MBG powders are 0.18, 0.24, 0.37 and 0.28, respectively; in other words, the I_1_/I_2_ value and the corresponding bioactivity followed the order 44% > 53% > 31% > 14%.

## 3. Discussion

In SP, the problem of inhomogeneity is frequently observed during the preparation of particles, including elemental particles (e.g., SrTiO_3_ [29] and CaMgSi_2_O_6_ [30]) because different precursors have different precipitation rates. In a previous study, we used TEOS, CN, and TEP as precursors (Si, Ca, and P precursors, respectively) to prepare SP-derived BG at a high calcination temperature of 700 °C, and the nanoparticles of amorphous SiO_2_ and the submicron particles of wollastonite-based glass were observed using TEM and X-ray energy-dispersive spectroscopy [31]. TEOS is insoluble with water because of its hydrolysis behavior, whereas CN and TEP are soluble in water, and the different precipitation behaviors of TEOS, CN, and TEP led to this inhomogeneity. In this study, TEOS, CN, and TEP were used to prepare MBG particles. However, no segregation or any second phase was detected in the XRD data (Figure 1) for any of the MBG particles (with treatments with various surfactant concentrations). This result implies that the hydrophobic PO groups and hydrophilic EO groups of the F127 surfactant attracted the insoluble TEOS and soluble CN and TEP during heating to avoid the problem of phase separation.

Two morphologies, smooth (Type I) and wrinkled (Type II) spheres, were observed. The Type I particles were observed frequently and were described in-depth in our previous studies [17,25]. A possible explanation for the formation of Type II particles is that the concentration of the partial droplets of the surfactant exceeded the CMC, causing the particles to aggregate to produce spherical micelles, elongated tubes, and stacked lamellae at the first, second, and third CMCs, respectively [22]. After SP, these tubes and stacked lamellae burned out, and the wrinkled mesoporous spheres were obtained. According to statistical analysis, the population of Type II particles for the 14 wt %, 31 wt %, 44 wt %, and 53 wt % F127-treated MBG powders were 0.0%, 0.0%, 44.6%, and 96.1% (the population of Type I particles were 100.0%, 100.0%, 55.4%, and 3.9%), respectively. This statistical result showed that (i) the CMC of F127 in this study was between 33 and 44 wt % and (ii) the population (%) of Type II particles increased with an increase in the surfactant concentration. In summary, the population of wrinkled spherical particles was closely related to the surfactant concentration.

The statistical analysis suggested a direct relation between particle size and surfactant concentration; the higher the surfactant concentration, the larger the particle size. It is well known that the particle size is a function of precursor, solid content in the precursor solution, and ultrasonic frequency [32]. In this study, the solid content in the precursor solution and ultrasonic frequency were fixed. The result suggested that the precursor solution of a high surfactant concentration provides more porosity to form larger particles than a lower surfactant concentration, which is supported by TEM results (Figure 3). In addition, there was no considerable correlation between particle size and particle morphology; the average particle sizes and their standard deviations for Type I and II particles were 1.12 ± 0.66 and 0.85 ± 0.66 µm, respectively. This indicates the distribution of elongated tubes and stacked lamellae was homogenous.

Furthermore, a detailed examination showed that all MBG powders exhibited a normal size distribution (submicron size) and not the bimodal distribution (nano and submicron size). A normal size distribution implied that the particle formation mechanism involved only “one-particle-per-drop” and not “gas-to-particle conversion” [33]. Furthermore, for the case of spray pyrolyzed BG (without addition of surfactant) [26], the effects of phase separation on loss of bioactivity were investigated. This phase-separated BG powder contains the submicron glass ceramic particles, with the amorphous phases and the minor phases of monoclinic wollastonite (JCPDS of 43-1460) and triclinic wollastonite (JCPDS 29-0372), and the amorphous SiO_2_ nanoparticles. These wollastonite phases reduce the amorphous region, which is responsible for bioactivity; therefore, the phase separation deteriorates the bioactivity of BG. So, the formation of amorphous SiO_2_ nanoparticles inhibits the formation of HA. This result supports that the view that the addition of a surfactant stabilizes the Si precursor and avoids the formation of amorphous SiO_2_ nanoparticles [31] that cause the gas-to-particle conversion for phase separation.

**Figure 6:**
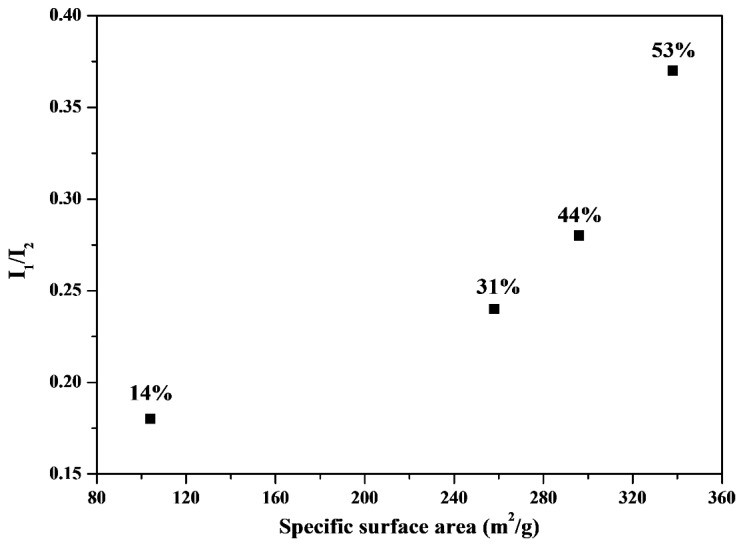
Comparison of I_1_/I_2_ (bioactivity) and surface area for various surfactant concentrations treated MBG. The values of surfactant concentration are given.

Although a high specific surface area of MBGs leads to a high bioactivity [7], the data of specific surface area must be compared with those of the bioactivity test to determine the relationship between bioactivity and specific surface area. Figure 6 shows the relationship between HA bioactivity (the I_1_/I_2_ value) and the corresponding specific surface area. The I_1_/I_2_ value (*i.e.*, bioactivity) increased with an increase in the specific surface area. The BET data suggested that the specific surface area of the MBG powders followed the order 44% (338.9 m^2^/g) > 53% (296.6 m^2^/g) > 31% (258.3 m^2^/g) > 14% (104.2 m^2^/g), which is consistent with the bioactivity order (I_1_/I_2_)) (44% (0.37) > 53% (0.28) > 31% (0.24) > 14% (0.18)). Therefore, maximizing the specific surface area is critical for bioactivity improvement in SP-derived MBG particles.

**Figure 7:**
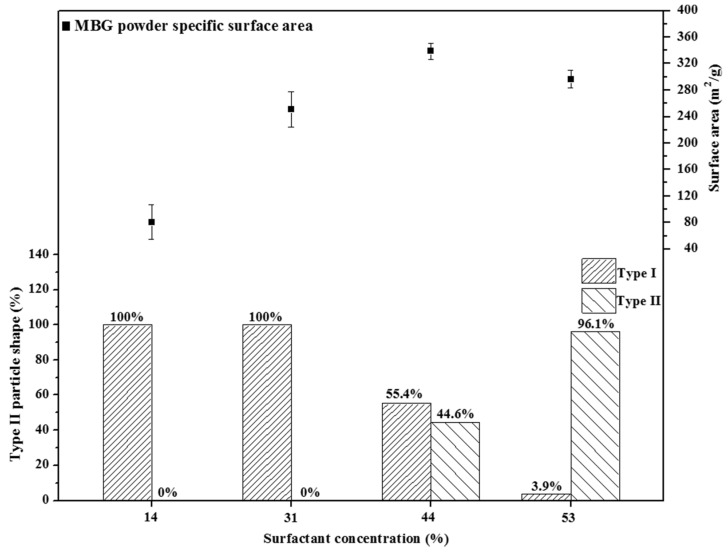
Relationship between particle shape and surface area for various surfactant concentrations treated MBG powders. The percentage values of Type I and II are given.

Previous studies have reported that the specific surface area of an MBG is determined by its pore size [5] and particle morphology [17]. First, we considered the case of MBG powders with a similar pore size to minimize the influence of pore size on the specific surface area. For example, 14 wt %, 31 wt %, and 44 wt % F127-treated MBG powders had a similar pore size of 7 nm, and the specific surface area increased with an increase in the surfactant concentration (104.2, 258.3, and 338.9 m^2^/g for 14 wt %, 31 wt %, and 44 wt %, respectively), implying that a MBG powder with a high surfactant concentration provides more micelle tubes, resulting in a high specific surface area (*i.e.*, a high density of the mesopores). Furthermore, in principle, a 53 wt % F127-treated MBG powder should contain the highest amount of micelle tubes and the largest specific surface area. However, the BET value of the 53 wt % F127-treated MBG powder was lower than that of the 44 wt % F127-treated MBG powder. A possible explanation is that in the case of 53 wt % F127-treated MBG powder, in precursor droplets, the surfactant concentration is higher than its CMC (between 33 wt % and 44 wt %), causing the micelles to aggregate and produce stacked lamellae and then settle on the droplets because of a lower density than water. After sintering, the organic moieties of these lamellae are burned to form the wrinkled structure (Type II). Figure 7 shows the particle morphology as a function of the surfactant concentration. The population of Type II morphology increased from 44.6% to 96.1% when the surfactant concentration was increased from 44 wt % to 53 wt %. This wrinkled structure may be attributable to the loss of the specific surface area because the structure reduced the mesopore density on the MBG surface (Figure 3d).

In summary, a suitable surfactant concentration that provides a high mesopore density and avoids the formation of aggregated surfactant lamellae must be determined for maximizing the specific surface area and in turn obtaining high bioactivity in MBGs.

## 4. Materials and Methods

### 4.1. Synthesis

The SiO_2_-CaO-P_2_O_5_ MBG powders were prepared from four F127 surfactant concentrations of 14 wt %, 31 wt %, 44 wt % and 53 wt %, using SP. Initially, the Si, Ca, and P precursors are 6.70 g tetraethyl orthosilicate (TEOS, Si(OC_2_H_5_)_4_, 99.9 wt %, Showa, Osaka, Japan), 1.40 g calcium nitrate tetrahydrate (CN, Ca(NO_3_)_2_ 4H_2_O, 98.5 wt %, Showa, Osaka, Japan) and 0.73 g triethyl phosphate (TEP, (C_2_H_5_)_3_PO_4_, 99 wt %, Alfa Aesar, Haverhill, MA, USA), respectively for the Si:Ca:P molar ratio of 80:15:5. Then, these precursors were mixed with 1.44, 3.97, 6.95, and 9.97 g F127 surfactants (for surfactant concentrations of 14 wt %, 31 wt %, 44 wt % and 53 wt %), dissolved in 1.00 g 0.5 M HCl and 60.00 g ethanol and stirred at room temperature for 24 h to form precursor solutions. For the SP process, the 10 mL of precursor solution was mixed with 90 mL of DI water to disperse into fine droplets using the nebulizer (King Ultrasonics Co., New Taipei, Taiwan) at the frequency of 1.65 MHz. Subsequently, the droplets formed particles in a tube furnace (D110, Dengyng, New Taipei, Taiwan), which has three preheating, calcination, and cooling zones of 400, 700, and 500 °C. The surfaces of the particles were then charged by electrons released from tungsten corona wire at high voltage (16 kV). Finally, the negatively charged powders were neutralized and condensed in an earthed stainless steel collector.

### 4.2. Characterization

In order to understand the detailed structures of MBG powders prepared using various F127 concentrations (14, 31, 44 and 53 wt %), the data of phase composition, surface structure, geometry, and specific surface area were examined using XRD, SEM, TEM and BET, respectively. Firstly, the phase composition was observed using the X-ray diffractometer (D2 Phaser, Billerica, MA, USA) with the operating voltage of 30 kV, the operating current of 10mA, the scan range of 2θ angle from 20° to 50° and the scan rate of 1° s^−1^. Secondly, the surface morphology was observed by using field-emission SEM (JSM-6500F, JEOL, Tokyo, Japan), and, for each MBG powder, over 300 MBG particles from a few SEM micrographs have been acquired to obtain the corresponding particle size distribution. Thirdly, field-emission TEM (Tecnai G2 F20, FEI, Hillsboro, OR, USA) was carried out to exam the detailed geometries and pore sizes of MBG; more than 100 pores were used to obtain the average value and standard deviation of pore size. Finally, the specific surface areas of various MBG powders were measured by the Brunauer-Emmett-Teller (BET) method with nitrogen adsorption and desorption isotherms under the temperature of −196 °C; the powders were degassed at 400 °C for 3 h before the BET measurements.

In addition, the test solution of Kokubo’s simulated body fluid (SBF) [9] has been previously used for *in vitro* bioactivity tests. The MBG powders were immersed in SBF at 37 °C for 1 day. The amount of SBF was adjusted to the specific surface areas of MBG. Solid to liquid ratio of 2 mg to 10 mL for the case of 14 wt %, 2 mg to 25 mL for the case of 31 wt %, 2 mg to 33 mL for the case of 44 wt % and 2 mg to 29 mL for the case of 53 wt %. Then, the immersed powers were dried at 100 °C for 1 day, and the phase composition of immersed powders was characterized using XRD to examine the bioactivity of MBG, the HA formation ability of MBG in SBF. The quantitative values of bioactivity of MBG powders were determined using the peak area from the largest peak of HA.

## 5. Conclusions

MBG powders treated with various F127 surfactant concentrations (14, 31, 44, and 53 wt %) were successfully synthesized using SP. The XRD patterns showed that all MBG powders were amorphous and without phase separation. In addition, two morphologies, smooth (Type I) and wrinkled (Type II) spheres, were detected using SEM and TEM. The population of Type II particles is a function of the surfactant concentration because, when the surfactant concentration was higher than its CMC, aggregated micelles were formed, in turn forming wrinkled spherical particles. Furthermore, more surfactants produced more micelles in precursor droplets, and the porosity increased after calcination, resulting in a high specific surface area. However, the MBG powder treated with the highest surfactant concentration (53 wt %) did not exhibit the highest specific surface area because the wrinkled structure reduced the mesopore density. Moreover, *in vitro* bioactive tests suggested that the order of HA formation rate was consistent with that of the specific surface area. Finally, a suitable surfactant concentration must be determined to obtain the maximum bioactivity of SP-derived MBG powders.
